# Clinicopathological significance of intratumoral and peritumoral lymphocytes and lymphocyte score based on the histologic subtypes of cutaneous melanoma

**DOI:** 10.18632/oncotarget.14736

**Published:** 2017-01-19

**Authors:** Cheol Keun Park, Sang Kyum Kim

**Affiliations:** ^1^ Department of Pathology, Severance Hospital, Yonsei University College of Medicine, Seoul, Republic of Korea

**Keywords:** melanoma, tumor infiltrating lymphocyte, lymphocytic score, peritumoral lymphocyte, intratumoral lymphocyte

## Abstract

The presence of tumor infiltrating lymphocytes is a favorable prognostic factor in cutaneous melanoma, but their clinicopathological significance in the intratumoral compartment compared to the peritumoral compartment is unclear. We investigated the clinicopathologic significance of tumor-infiltrating lymphocytes and lymphocyte score in intra- and peritumoral compartments in 177 Korean patients who had undergone surgical excision of cutaneous melanoma. No significant correlation was observed between various clinicopathologic factors and the presence of intratumoral lymphocytes. However, high peritumoral lymphocyte scores were associated with lower Clark levels (*P* = 0.001), shallower Breslow thicknesses (*P* = 0.006), and fewer mitotic counts (*P* = 0.01) than tumors with lower scores. There was a trend for longer disease-free survival in cases with peritumoral lymphocytes (*P* = 0.07) than those without peritumoral lymphocytes. In patients with acral lentiginous melanoma, a strong association between a high peritumoral lymphocyte score and shallow Clark level was apparent (*P* = 0.03), and the presence of peritumoral lymphocytes (*P* = 0.02) and a high intratumoral lymphocyte score (*P* = 0.04) was also associated with longer disease-free survival. Particularly, low intratumoral lymphocyte score remarkably affected tumor recurrence and distant metastasis in a multivariate analysis using Cox regression test (H.R. = 0.304, 95% C.I. = 0.078–1.185, *P* = 0.09). Thus, the presence of lymphocytes and high lymphocyte scores in the intratumoral and peritumoral compartments are valid prognostic factors in cutaneous melanoma.

## INTRODUCTION

Malignant transformation of tissue destroys its normal structure and leads to an immune response that includes infiltration of the tissue with immune cells that target tumor cells [1]. Immune cell infiltration is an important protective mechanism and forms the basis for adoptive cell therapy in cancer treatment. The use of adoptive cell therapy in patients with cutaneous melanoma is particularly promising. The therapy entails initial lympho-depletion followed by administration of *ex vivo* expanded tumor infiltrating lymphocytes (TILs) and large doses of interleukin 2 (IL-2) and had an approximately 50% response rate in patients with highly advanced and metastatic melanoma [2, 3]. Perhaps more important, it was effective in patients who had responded poorly to existing immumotherapies, such as treatment modalities based on IL-2 [4]. Adoptive cell therapy has been applied to cases of metastatic melanoma or lung cancer in which tumor cells evaded the immune cell attack in a process referred to as immunoediting [5]. Researchers sought to counteract immunoediting by reactivating the immune response after its inhibition by the tumor cells [3, 4, 6–8].

The application of immune cell infiltration in the treatment of cutaneous melanoma is increasing, despite some uncertainty as to its efficacy [9, 10]. Most studies have been of a small number of cases or did not include the identification of the type of infiltrating immune cells [11]. This has resulted in an uncertain prognosis for patients receiving the treatment. This uncertainty was addressed in a recent study of a large series of patients, in which it was observed that marked accumulation of TILs was a favorable prognostic factor [12]. Also, in a study sponsored by The Cancer Genome Atlas project, cutaneous melanoma patients with a higher lymphocyte score (LS) had longer overall survival than patients with a lower LS, regardless of the histological subtype [13]. While these studies are promising, caution is still appropriate, as in these large-series studies, the location of the lymphocytes, whether in the intratumoral and peritumoral compartments, was not always determined. Therefore, the location of the infiltrating lymphocytes may be an important consideration.

It may also be important to consider the efficacy of TILs in melanomas of different histological subtypes. Generally, superficial spreading melanoma (SSM) is associated with favorable prognosis, while nodular melanoma (NM) has the poorest prognosis among cutaneous melanoma subtypes in the United States and East Asia [14, 15]. However, the effect of tissue on patient prognosis is uncertain and controversial for some cutaneous melanoma subtypes, including acral lentiginous melanoma (ALM). ALM usually occurs in areas that are rarely exposed to the sun, such as acral or mucosal sites and is associated with *KIT* mutations [16]. According to United States Surveillance, Epidemiology, and End Results (SEER) data [17], patients with ALM have poor prognoses compared to patients with other histologic subtypes of melanoma, whereas it follows an indolent course in a small number of patients studied in Korea [18].

In this study, we have evaluated the intratumoral and peritumoral compartments of cutaneous melanomas for the presence and number of TILs and assessed their correlation with various clinicopathological factors, including histological subtype. We related these parameters to prognostic significance.

## RESULTS

### Basic clinicopathological features of cutaneous melanoma

The clinicopathological features of the 177 cutaneous melanoma cases studied were summarized in Table 1. Among these, there were 80 cases of ALM, 68 cases of NM, and 29 cases of SSM. Compared to the other subtypes of cutaneous melanoma, NM had a higher Clark level (*P* < 0.001), greater Breslow thickness (*P* < 0.001), more ulceration (*P* < 0.001), a higher mitotic count (*P* < 0.001), more lymphovascular invasion (*P* = 0.01), and more sentinel lymph node metastasis (*P* < 0.001).

**Table 1:** Clinicopathological characteristics of 177 invasive cutaneous melanomas according to the histologic subtype

Variables	Case No.	ALM		NM		SSM		*P* value
		*n* = 80 (%)		*n* = 68 (%)		*n* = 29 (%)		
Age								
< 60 years	95	39	(48.8)	39	(57.4)	17	(58.6)	0.49
≥ 60 years	82	41	(51.3)	29	(42.6)	12	(41.4)	
Gender								
Male	86	41	(51.3)	34	(50.0)	11	(37.9)	0.45
Female	91	39	(48.8)	34	(50.0)	18	(62.1)	
SLNM^a^								
Absent	98	55	(85.9)	29	(50.0)	14	(93.3)	< 0.001
Present	39	9	(14.1)	29	(50.0)	1	(6.7)	
Recurrence / Metastasis								
Absent	131	64	(80.0)	41	(60.3)	26	(89.7)	0.003
Present	46	16	(20.0)	27	(39.7)	3	(10.3)	
Survival								
Alive	136	65	(81.3)	43	(63.2)	28	(96.6)	0.001
Expired	41	15	(18.8)	25	(36.8)	1	(3.4)	
Follow-up period, mo^b^		40	(47.5)	21	(28.3)	36	(44.0)	0.001
Clark level								
II or III	47	27	(33.8)	4	(5.9)	16	(55.2)	< 0.001
IV or V	130	53	(66.3)	64	(94.1)	13	(48.8)	
Breslow thickness								
≤ 1.00 mm	48	25	(31.3)	3	(4.4)	20	(69.0)	< 0.001
1.01–2.00 mm	47	27	(33.8)	12	(17.6)	8	(27.6)	
2.01–4.00 mm	33	17	(21.3)	15	(22.1)	1	(3.4)	
> 4.00 mm	49	11	(13.8)	38	(55.9)	0	(0.0)	
Ulceration								
Absent	126	58	(72.5)	39	(57.4)	29	(100)	< 0.001
Present	51	22	(27.5)	29	(42.6)	0	(0.0)	
Mitosis^c^								
≤ 5/10 HPFs	122	63	(78.8)	33	(48.5)	26	(89.7)	< 0.001
> 5/10 HPFs	55	17	(21.3)	35	(51.5)	3	(10.3)	
LVI								
Absent	162	76	(95.0)	57	(83.8)	29	(100)	0.01
Present	15	4	(5.0)	11	(16.2)	0	(0.0)	
Mutation status^d^								
*BRAF* mutant	12	2	(11.8)	10	(43.5)	0	(0.0)	0.06
*BRAF* wild	30	15	(88.5)	13	(56.5)	2	(100)	

ALM, acral lentiginous melanoma; NM, nodular melanoma; SSM, superficial spreading melanoma; SLNM, sentinel lymph node metastasis; LVI, lymphovascular invasion; mo, month.

^a^SLNM was evaluated in 137 cases.

^b^Follow-up period was presented as median (interquartile range).

^c^Mean mitotic count of 177 cases were 5.78/10HPFs.

^d^Mutation study was performed in 42 cases. Two *NRAS* mutant cases belonged to *BRAF* wild group.

### Lymphocyte and lymphocyte score status in all cases studied

**Figure 1:**
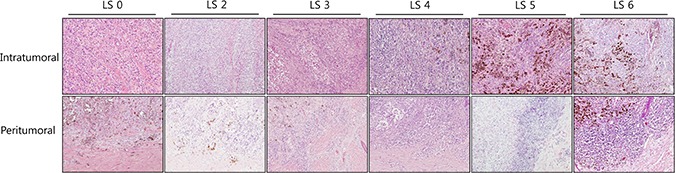
Microscopic examples of low and high lymphocyte scores in the intratumoral and peritumoral compartments of cutaneous melanomas Representative microphotographs of the intratumoral and peritumoral lymphocyte scores with original magnification ×100 (H&E).

TILs were identified as associated with either the location of lymphocytes, whether intratumoral or peritumoral, and the LS for each population was calculated (Table 2). Sixty-six patients (37.29%) had intratumoral lymphocytes and 142 (82.23%) had peritumoral lymphocytes. High intratumoral lymphocyte score (I-LS) were observed for 27 patients (15.25%) and high peritumoral lymphocyte score (P-LS) were observed for 76 cases (42.94%). The representative microscopic photographs were presented in Figure 1.

**Table 2:** Clinicopathological characteristics of 177 invasive cutaneous melanomas according to lymphocyte and lymphocyte score status of intratumoral and peritumoral compartment

Variables	Peritumoral lymphocyte			Peritumoral lymphocyte score			Intratumoral lymphocyte			Intratumoral lymphocyte score		
	Present (%)	Absent (%)	*P*-value	High (%)	Low (%)	*P*-value	Present (%)	Absent (%)	*P*-value	High (%)	Low (%)	*P*-value
	(*n* = 142)	(*n* = 35)		(*n* = 76)	(*n* = 101)		(*n* = 66)	(*n* = 111)		(*n* = 27)	(*n* = 150)	
Age												
< 60 years	77 (54.2)	18 (51.4)	0.77	40 (52.6)	55 (54.5)	0.81	28 (42.4)	67 (60.4)	0.02	10 (37.0)	85 (56.7)	0.06
≥ 60 years	65 (45.8)	17 (48.6)		36 (47.4)	46 (45.5)		38 (57.6)	44 (39.6)		17 (63.0)	65 (43.3)	
Gender												
Male	66 (46.5)	20 (57.1)	0.26	41 (53.9)	45 (44.6)	0.22	36 (54.5)	50 (45.0)	0.22	18 (66.7)	68 (45.3)	0.04
Female	76 (53.5)	15 (42.9)		35 (46.1)	56 (55.4)		30 (45.5)	61 (55.0)		9 (33.3)	82 (54.7)	
Histologic subtype												
ALM	62 (43.7)	18 (51.4)	0.36	36 (47.4)	44 (43.6)	0.17	33 (50.0)	47 (42.3)	0.43	15 (55.6)	65 (43.3)	0.47
NM	54 (38.0)	14 (40.0)		24 (31.6)	44 (43.6)		25 (37.9)	43 (38.7)		9 (33.3)	59 (39.3)	
SSM	26 (18.3)	3 (8.6)		16 (21.1)	13 (12.9)		8 (12.1)	21 (18.9)		3 (11.1)	26 (17.3)	
Clark level												
II or III	42 (29.6)	5 (14.3)	0.07	30 (39.5)	17 (16.8)	0.001	16 (24.2)	31 (27.9)	0.59	9 (33.3)	38 (25.3)	0.39
IV or V	100 (70.4)	30 (85.7)		46 (60.5)	84 (83.2)		50 (75.8)	80 (72.1)		18 (66.7)	112 (74.7)	
Breslow thickness												
≤ 1.00 mm	42 (29.6)	6 (17.1)	0.16	28 (36.8)	20 (19.8)	0.006	15 (22.7)	33 (29.7)	0.42	9 (33.3)	39 (26.0)	0.44
1.01–2.00 mm	40 (28.2)	7 (20.0)		23 (30.3)	24 (23.8)		22 (33.3)	25 (22.5)		8 (29.6)	39 (26.0)	
2.01–4.00 mm	25 (17.6)	8 (22.9)		13 (17.1)	20 (19.8)		11 (16.7)	22 (19.8)		6 (22.2)	27 (18.0)	
> 4.00 mm	35 (24.6)	14 (40.0)		12 (15.8)	37 (36.6)		18 (27.3)	31 (27.9)		4 (14.8)	45 (30.0)	
Ulceration												
Absent	103 (72.5)	23 (65.7)	0.43	58 (76.3)	68 (67.3)	0.19	41 (62.1)	85 (76.6)	0.04	20 (74.1)	106 (70.7)	0.72
Present	39 (27.5)	12 (34.3)		18 (23.7)	33 (32.7)		25 (37.9)	26 (23.4)		7 (25.9)	44 (29.3)	
Mitosis^a^												
≤ 5/10 HPFs	98 (69.0)	24 (68.6)	0.96	60 (78.9)	62 (61.4)	0.01	48 (72.7)	74 (66.7)	0.40	22 (81.5)	100 (66.7)	0.13
> 5/10 HPFs	44 (31.0)	11 (31.4)		16 (21.1)	39 (38.6)		18 (27.3)	37 (33.3)		5 (18.5)	50 (33.3)	
LVI												
Absent	133 (93.7)	29 (82.9)	0.04	70 (92.1)	92 (91.1)	0.81	59 (89.4)	103 (92.8)	0.43	25 (92.6)	137 (91.3)	0.99
Present	9 (6.3)	6 (17.1)		6 (7.9)	9 (8.9)		7 (10.6)	8 (7.2)		2 (7.4)	13 (8.7)	
SLNM^b^												
Absent	78 (71.6)	20 (71.4)	0.99	41 (75.9)	57 (68.7)	0.36	38 (67.9)	60 (74.1)	0.43	15 (65.2)	83 (72.8)	0.46
Present	31 (28.4)	8 (28.6)		13 (24.1)	26 (31.3)		18 (32.1)	21 (25.9)		8 (34.8)	31 (27.2)	
Mutation status^c^												
*BRAF* mutant	11 (35.5)	1 (9.1)	0.13	6 (33.3)	6 (25.0)	0.55	7 (38.9)	5 (20.8)	0.20	3 (60.0)	9 (24.3)	0.13
*BRAF* wild	20 (64.5)	10 (90.9)		12 (66.7)	18 (75.0)		11 (61.1)	19 (79.2)		2 (40.0)	28 (75.7)	
Recurrence /Metastasis												
Absent	109 (76.8)	22 (62.9)	0.09	60 (78.9)	71 (70.3)	0.19	50 (75.8)	81 (73.0)	0.68	23 (85.2)	108 (72.0)	0.15
Present	33 (23.2)	13 (37.1)		16 (21.1)	30 (29.7)		16 (24.2)	30 (27.0)		4 (14.8)	42 (28.0)	
Survival												
Alive	110 (77.5)	26 (74.3)	0.69	60 (78.9)	76 (75.2)	0.56	52 (78.8)	84 (75.7)	0.64	21 (77.8)	115 (76.7)	0.90
Expired	32 (22.5)	9 (25.7)		16 (21.1)	25 (24.8)		14 (21.2)	27 (24.3)		6 (22.2)	35 (23.3)	

ALM: acral lentiginous melanoma; NM: nodular melanoma; SSM: superficial spreading melanoma; HPF: high-power filed; LVI: lymphovascular invasion; SLNM: sentinel lymph node metastasis.

^a^Mean mitotic count of 177 cases were 5.78/10HPFs.

^b^SLNM was Evaluated in 137 cases.

^c^Mutation study was performed in 42 cases. Two *NRAS* mutant cases belonged to *BRAF* wild group.

Considering the intratumoral compartment, there were significant correlations between the presence of intratumoral lymphocytes and various clinical factors: the age of the patient at the time of surgery, the sex of the patient and incidence of ulceration (*P* < 0.05 for all). Meanwhile, there were no significant differences in pathologic factors according to the presence of intratumoral lymphocytes.

In contrast, there were significant correlations between the presence of peritumoral lymphocytes and various pathologic factors. Cases with high P-LS had lower Clark levels and shallower Breslow thickness than cases with low P-LS in histologic examination (*P* = 0.001 and *P* = 0.006, respectively). This finding is consistent with the suggestion that high P-LS were present in the early stages of invasive cutaneous melanoma. In details, patients with high P-LS also had fewer mitotic counts (≤ 5 per 10 high-power fields) than patients with low P-LS (*P* = 0.01). There was an association between the presence of peritumoral lymphocytes and absence of lymphovascular invasion (*P* = 0.04) and less recurrence or metastasis (*P* = 0.09), although clinical follow-up data did not reveal a correlation between P-LS and tumor recurrence, distant metastasis or patients’ survival.

### Lymphocyte status and lymphocyte score status in each histological subtype

Next, we analyzed clinicopathologic features according to lymphocytic infiltration in major histological subtypes of cutaneous melanoma. There were no significant differences with respect to location of the lymphocytes and the parameters measured in either NM or SSM (*P* > 0.05 for all; Supplementary Tables 1, 2, 3, 4 in the Supplementary Data).

**Table 3:** Clinicopathological characteristics of 80 acral lentiginous melanomas according to lymphocyte and lymphocyte score status of intratumoral and peritumoral compartment

Variables	Peritumoral lymphocyte			Peritumoral lymphocyte score			Intratumoral lymphocyte			Intratumoral lymphocyte score		
	Present (%)	Absent (%)	*P*-value	High (%)	Low (%)	*P*-value	Present (%)	Absent (%)	*P*-value	High (%)	Low (%)	*P*-value
	(*n* = 62)	(*n* = 18)		(*n* = 36)	(*n* = 44)		(*n* = 33)	(*n* = 47)		(*n* = 15)	(*n* = 65)	
Age												
< 60 years	30 (48.4)	9 (50.0)	0.90	15 (41.7)	24 (54.5)	0.25	12 (36.4)	27 (57.4)	0.07	5 (33.3)	34 (52.3)	0.19
≥ 60 years	32 (51.6)	9 (50.0)		21(58.3)	20(45.5)		21 (63.6)	20 (42.6)		10 (66.7)	31 (47.7)	
Gender												
Male	29(46.8)	12(66.7)	0.14	22 (61.1)	19 (43.2)	0.11	18 (54.5)	23 (48.9)	0.62	10 (66.7)	31 (47.7)	0.19
Female	33 (53.2)	6 (33.3)		14 (38.9)	25 (56.8)		15 (45.5)	24 (51.1)		5 (33.3)	34 (52.3)	
Clark level												
II or III	23 (37.1)	4 (22.2)	0.24	17 (47.2)	10 (22.7)	0.03	10 (30.3)	17 (36.2)	0.59	8 (53.3)	19 (29.2)	0.08
IV or V	39 (62.9)	14 (77.8)		19 (52.8)	34 (77.3)		23 (69.7)	30 (63.8)		7 (46.7)	46 (70.8)	
Breslow thickness												
≤ 1.00 mm	22 (35.5)	3 (16.7)	0.35	16 (44.4)	9 (20.5)	0.07	8 (24.2)	17 (36.2)	0.68	6 (40.0)	19 (29.2)	0.36
1.01–2.00 mm	21 (33.9)	6 (33.3)		11 (30.6)	16 (36.4)		13 (39.4)	14 (29.8)		5 (33.3)	22 (33.8)	
2.01–4.00 mm	11 (17.7)	6 (33.3)		7 (19.4)	10 (22.7)		7 (21.2)	10 (21.3)		4 (26.7)	13 (20.0)	
> 4.00 mm	8 (12.9)	3 (16.7)		2 (5.6)	9 (20.5)		5 (15.2)	6 (12.8)		0 (0.0)	11 (16.9)	
Ulceration												
Absent	46 (74.2)	12 (66.7)	0.56	25 (69.4)	33 (75.0)	0.58	18 (54.5)	40 (85.1)	0.005	9 (60.0)	49 (75.4)	0.34
Present	16 (25.8)	6 (33.3)		11 (30.6)	11 (25.0)		15 (45.5)	7 (14.9)		6 (40.0)	16 (24.6)	
Mitosis^a^												
≤ 5/10 HPFs	48 (77.4)	15 (83.3)	0.75	29 (80.6)	34 (77.3)	0.72	26 (78.8)	37 (78.7)	0.99	13 (86.7)	50 (76.9)	0.41
> 5/10 HPFs	14 (22.6)	3 (16.7)		7 (19.4)	10 (22.7)		7 (21.2)	10 (21.3)		2 (13.3)	15 (23.1)	
LVI												
Absent	60 (96.8)	16 (88.9)	0.22	34 (94.4)	44 (95.5)	> 0.99	32 (97.0)	44 (93.6)	0.64	15 (100)	61 (93.8)	> 0.99
Present	2 (3.2)	2 (11.1)		2 (5.6)	2 (4.5)		1 (3.0)	3 (6.4)		0 (0.0)	4 (6.2)	
SLNM^b^												
Absent	42 (96.8)	13 (96.8)	0.67	24 (82.8)	31 (88.6)	0.72	24 (80.0)	31 (91.2)	0.29	10 (71.4)	45 (90.0)	0.10
Present	8 (3.2)	1 (3.2)		5 (17.2)	4 (11.4)		6 (20.0)	3 (8.8)		4 (28.6)	5 (10.0)	
Mutation status^c^												
*BRAF* mutant	2 (18.2)	0 (0.0)	0.52	1 (14.3)	1 (10.0)	> 0.99	2 (28.6)	0 (0.0)	0.15	0 (0.0)	2 (12.5)	> 0.99
*BRAF* wild	9 (81.8)	6 (100)		6 (85.7)	9 (90.0)		5 (71.4)	10 (100)		1 (100)	14 (87.5)	
Recurrence / Metastasis												
Absent	53 (85.5)	11 (61.1)	0.04	30 (83.3)	34 (77.3)	0.50	29 (87.9)	35 (74.5)	0.14	15 (100)	49 (75.4)	0.03
Present	9 (14.5)	7 (38.9)		6 (16.7)	10 (22.7)		4 (12.1)	12 (25.5)		0 (0.0)	16 (24.6)	
Survival												
Alive	51 (82.3)	14 (77.8)	0.73	29 (80.6)	36 (81.8)	0.89	28 (84.8)	37 (78.7)	0.49	13 (86.7)	52 (80.0)	0.72
Expired	11 (17.7)	4 (22.2)		7 (19.4)	8 (18.2)		5 (15.2)	10 (21.3)		2 (13.3)	13 (20.0)	

HPF: high-power field; LVI: lymphovascular invasion; SLNM: sentinel lymph node metastasis.

^a^Mean mitotic count of 177 cases were 5.78/10HPFs.

^b^SLNM was Evaluated in 62 cases.

^c^Mutation study was performed in 17 cases. One NRAS mutant case belonged to BRAF wild group

**Table 4:** Multivariate analysis of 177 invasive cutaneous melanomas of the impact of variable linicopathologic factors on recurrence/metastasis and survival

Category	Variables	Recurrence/Metastasis		Survival	
		HR (95% CI)	*P*-value	HR (95% CI)	*P*-value
Age	< 60 years	1		1	
	≥ 60 years	0.939 (0.435–2.027)	0.87	1.949 (0.863–4.403)	0.11
Gender	Female	1		1	
	Male	2.354 (1.044–5.304)	0.04	2.552 (1.071–6.078)	0.03
Histologic subtype	ALM	1		1	
	NM	2.209 (0.825–5.914)	0.12	1.374 (.438–4.303)	0.59
	SSM	0.718 (0.150–3.429)	0.68	N/A	N/A
Clark level	II or III	1		1	
	IV or V	1.608 (0.414–6.247)	0.49	1.508 (0.242–9.389)	0.66
Breslow thickness	≤ 1.00 mm	1		1	
	1.01–2.00 mm	1.193 (0.295–4.823)	0.80	0.949 (0.067–13.375)	0.97
	2.01–4.00 mm	0.471 (0.089–2.476)	0.37	4.505 (0.400–50.677)	0.22
	> 4.00 mm	0.640 (0.124–3.318)	0.60	6.344 (0.476–84.546)	0.16
Ulceration	Absent	1		1	
	Present	1.187 (0.539–2.614)	0.67	1.597 (0.684–3.730)	0.28
Mitosis	≤ 5/10 HPFs	1		1	
	> 5/10 HPFs	1.130 (0.464–2.749)	0.79	0.582 (0.240–1.411)	0.23
LVI	Absent	1		1	
	Present	1.026 (0.355–2.969)	0.96	0.947 (0.303–2.958)	0.93
SLNM	Absent	1		1	
	Present	2.525 (1.050–6.070)	0.04	2.196 (0.844–5.715)	0.11
Intratumoral lymphocyte	Absent	1		1	
	Present	1.638 (0.657–4.079)	0.29	0.772 (0.225–2.651)	0.68
Peritumoral lymphocyte	Absent	1		1	
	Present	0.548 (0.214–1.400)	0.21	0.817 (0.267–2.500)	0.72
I-LS	Low	1		1	
	High	0.304 (0.078–1.185)	0.09	1.855 (0.542–6.341)	0.33
P-LS	Low	1		1	
	High	0.927 (0.387–2.219)	0.87	0.809 (0.255–2.568)	0.72

HR: hazard ratio; CI: confidence interval; ALM: acral lentiginous melanoma; NM: nodular melanoma; SSM: superficial spreading melanoma; HPF: high-power filed; LVI: lymphovascular invasion; SLNM: sentinel lymph node metastasis; I-LS: intratumoral lymphocyte score; P-LS: peritumoral lymphocyte score; N/A: Not applicable.

However, in ALM, cases with high P-LS had lower Clark levels and tended to have shallower Breslow thickness (*P* = 0.03 and *P* = 0.07, respectively, Table 3). Among this subtype, cases with peritumoral lymphocytes had less recurrence and metastasis (*P* = 0.04), which is consistent with our findings when we considered all cases combined (Table 2). In the cases of ALM, we also noted more intratumoral lymphocytes in older patients and patients with ulceration (*P* = 0.07 and *P* = 0.005, respectively, Table 3) and cases with high I-LS were associated with less frequent recurrence or metastasis (*P* = 0.03).

### Clinicopathologic significance according to the combination of lymphocyte status or score in all cases studied and acral lentiginous melanoma

Among 177 cases, 60 presented with lymphocytes in both intra- and peritumoral compartments. To investigate the relationship between various clinicopathologic factors and lymphocytes in both compartments, the cases were divided into four groups (Supplementary Table 5 in the Supplementary Data): absence of lymphocytes in both compartments (29 cases, 16.4%), intratumoral lymphocytes only (6 cases, 3.4%), peritumoral lymphocytes only (82 cases, 46.3%), and lymphocytes in both compartments (60 cases, 33.9%). The absence of lymphocytes in both compartments was associated with a deeper Breslow thickness (*P* = 0.005). When ulceration was present, lymphocytic infiltration in both compartments was less likely compared with tumors without ulcerations (*P* = 0.02).

With respect to LS, the cases were divided into four subgroups (Supplementary Table 6 in Supplementary Data): Low LS in both compartments (92 cases, 51.9%), high LS only in the intratumoral compartment (9 cases, 5.1%), high LS only in the peritumoral compartment (58 cases, 32.8%), and high LS in both compartments (18 cases, 10.2%). Cases with high LS in both compartments or the peritumoral compartment alone had a significantly shallower Clark level and Breslow thickness (*P* = 0.009 and 0.003, respectively). In addition, these two subgroups with high P-LS tended toward lower mitotic counts (≤ 5 per 10 high-power fields) compared with other subgroups (*P* = 0.06).

We separately analyzed clinicopathologic features of ALM according to tumor compartments. Cases with lymphocytes in any compartment had a significantly lower incidence of recurrence or metastasis than other cases (Supplementary Tables 7, 8 in the Supplementary Data, *P* = 0.04). However, there were no significant correlations between LS and various clinicopathologic factors (Supplementary Table 8 in the Supplementary Data).

### Lymphocyte and mutation status

We also analyzed the correlation between lymphocyte infiltration and cutaneous melanoma mutation status. Among 177 cases, mutation study findings from the time of diagnosis were available for 42. The mutation studies performed were as follows: *BRAF* only (15 cases), *BRAF/KIT* (24 cases), and *BRAF/NRAS* (3 cases). Twelve and two cases had *BRAF* and *NRAS* mutations, respectively. The remaining 28 cases were negative for mutations.

*BRAF* mutations were frequently observed in patients with NMs (*P* = 0.06, Table 1). In addition, *BRAF* mutations tended to be associated with the presence of peritumoral lymphocytes, although this was not significant (*P* = 0.13, Table 2). There was no significant correlation between LS and mutation status for any histologic subtype (Table 3 and Supplementary Tables 1, 2 in the Supplementary Data).

### Survival analysis

**Figure 2:**
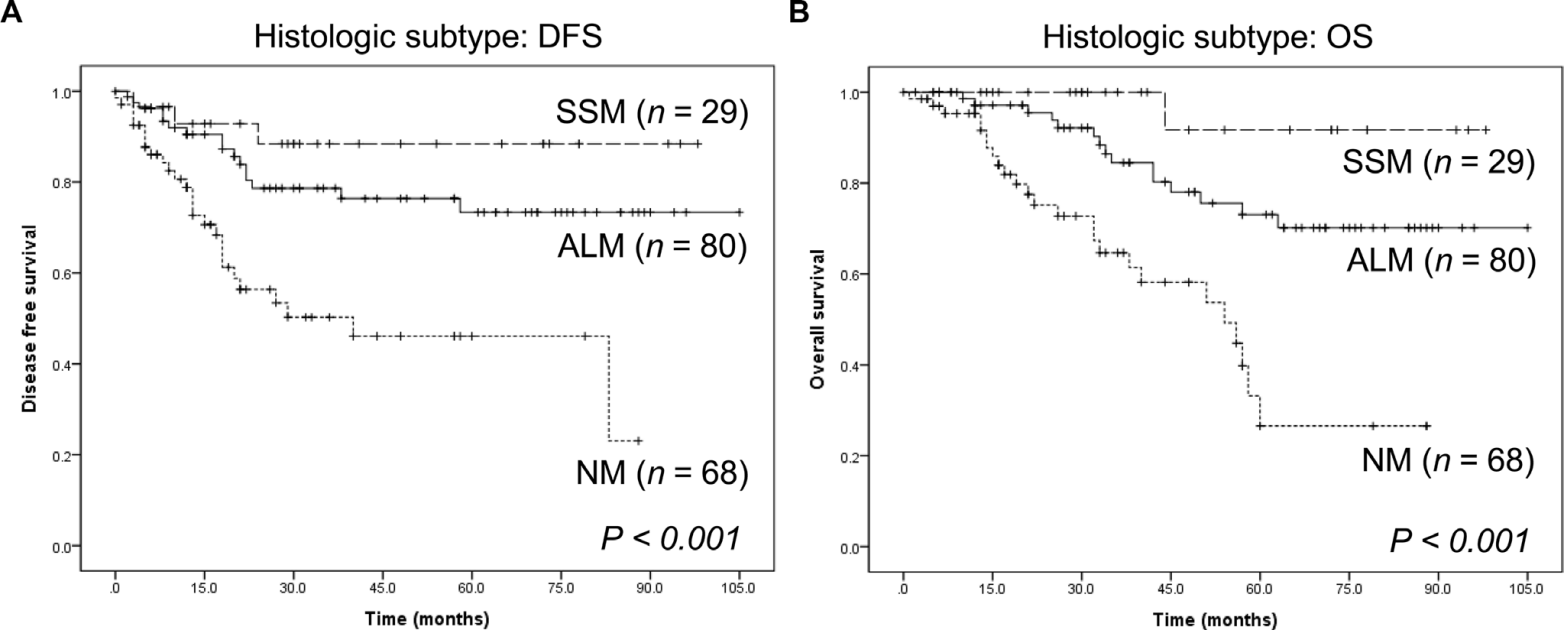
Survival of patients with cutaneous melanoma of different histological subtypes (**A**) Disease-free survival. (**B**) Overall survival. ALM: acral lentiginous melanoma; NM: nodular melanoma; SSM: superficial spreading melanoma.

All 177 patients were clinically followed up, and there were 46 incidences of loco-regional recurrence or systemic metastasis and 41 deaths (Table 1). In Kaplan-Meier analysis based on the histological subtype, NM had the poorest prognosis (Figure 2). The prognosis for patients with SSM was favorable with respect to disease-free survival (DFS) and overall survival (OS) (*P* < 0.001 for both).

**Figure 3:**
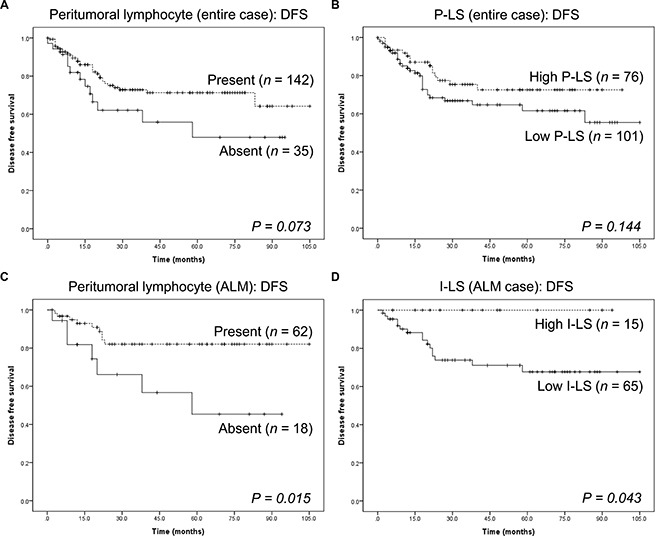
Kaplan-Meier survival analysis in intratumoral and peritumoral compartments (**A**) Disease-free survival of all cases of cutaneous melanomas in the study, in the presence or absence of peritumoral lymphocytes. (**B**) Disease-free survival of all cases of cutaneous melanoma in the study, compared to the peritumoral lymphocyte scores. (**C**) Disease-free survival of 80 acral lentiginous melanomas in the presence or absence of peritumoral lymphocytes. (**D**) Disease-free survival of 80 acral lentiginous melanomas compared to the intratumoral lymphocyte score.

Considering the group together, patients with peritumoral lymphocytes and a high P-LS tended to have longer DFS than those without peritumoral lymphocytes or low P-LS, but the differences were not significant (*P* = 0.07, Figure 3A and P = 0.14, Figure 3B, respectively). The presence or absence of intratumoral and peritumoral lymphocytes and their respective LS did not affect overall survival (*P* > 0.05 for all, Supplementary Figure 1 in the Supplementary Data).

When histological subtypes were compared, there was no significant association between NM, SSM, and ALM with respect to the presence of lymphocytes or LS and DFS and OS (*P* > 0.05 for all; Supplementary Figures 2–4 in the Supplementary Data). However, patients with ALM whose tumors had peritumoral lymphocytes and high I-LS had significantly longer DFS (*P* = 0.02, Figure 3C and P = 0.04, Figure 3D, respectively). We conclude that both peritumoral lymphocytes and high I-LS extended DFS in these patients.

For adjustment of parameters affecting a patient's survival, multivariable analysis was performed using Cox regression test (Table 4). When we assessed a total of 177 melanoma patients, male gender affected tumor recurrence and distant metastasis (H.R. = 2.354, 95% C.I. = 1.044–5.304, *P* = 0.04) and OS (H.R. = 2.552, 95% C.I. = 1.071–6.078, *P* = 0.03), respectively. Metastasis to a sentinel lymph node also affected tumor recurrence and distant metastasis (H.R. = 2.525, 95% C.I. = 1.050–6.071, *P* = 0.04). Although it was not statistically significant, low I-LS affected tumor recurrence and distant metastasis (H.R. = 0.304, 95% C.I. = 0.078–1.185, *P* = 0.09). When we analyzed patients’ prognosis by melanoma subtypes, any statistical significance was identified in multivariable analysis (Supplementary Table 9 in the Supplementary Data).

## DISCUSSION

TILs, produced by the immune system in response to the invasion by the tumor, are frequently observed in tumors, including those associated with cutaneous melanoma [19–21]. Their presence appears to be a favorable prognostic factor, regardless of the type of lymphocyte [22, 23], and they have been applied successfully in adoptive cell therapy [2]. However, reports on the prognostic impact and usefulness of TILs as a treatment modality have limitations, such as a small number of cases as well as inadequate consideration of both the lymphocyte subsets and the location of the lymphocyte, whether intratumoral or peritumoral [11].

Here, we analyzed a large series of invasive cutaneous melanoma and assessed the relationship between prognosis and the presence and location of the lymphocytes associated with the tumors. A newly developed parameter, LS, was used to assess the distribution and density of lymphocytes in the two compartments [12]. We believe this is the first report evaluating the effect of lymphocytes in the intratumoral and peritumoral compartments in invasive cutaneous melanoma.

We assessed the presence of lymphocytes and LS in each compartment in different histological subtypes of melanoma. We observed that the presence of peritumoral lymphocytes was a favorable prognostic factor when all cases were considered together. When the cases of ALM were considered separately, a high LS in either compartment tended to be favorable. However, in NM and SSM, the presence of lymphocytes in both compartments and their associated LS were not correlated with any clinicopathological factor and did not affect DFS or OS. Finally, we interpret our findings to indicate that high LS is a favorable prognostic factor in ALM but not in other subtypes, although a previous study identified immune signatures associated with improved survival independent of subtypes of cutaneous melanoma [13].

According to both the literature and our results, SSM has a favorable prognosis while NM and ALM have poor prognoses [15]. The differences in prognoses may be due to the type of dominant growth phase of the histological subtypes [24]. However, it is unknown how the location of the lymphocytes might affect the prognosis of patients with histologically different tumors.

When considering the importance of the lymphocyte location, it is possible that the presence of intratumoral or peritumoral lymphocyte and LS may be affected by histological or genetic subtype. For instance, the *NRAS* mutation has been more frequently detected in NM than in other subtypes [25, 26]. Activation of the oncogenic RAS pathway by the *NRAS* mutation suppresses the immune response by decreasing expression of major histocompatibility complex on tumor-cell surfaces and recruitment of regulatory T lymphocytes [27]. In a large-series study performed in the United States and Australia, when the *NRAS* mutation was present in melanoma, the tumor infiltrating lymphocyte grade was lower [28].

To our knowledge, this study is the first report on the prognostic impact of high LS in ALM. Although the mechanism is uncertain, we can assume that there was a lower incidence of the *BRAF* or *NRAS* mutations in these cases, which have poor prognoses [28, 29]. In addition, in our study, cases of ALM had more intratumoral lymphocytes and higher I-LS than other subtypes. Based on this finding, we suggest that high I-LS may increase DFS more in ALM than in the other subtypes.

The exact mechanism by which TILs can affect ALM prognosis has not been elucidated, but several hypotheses could explain our findings. Unlike ALM, other cutaneous melanoma subtypes frequently present ultraviolet signatures and harbor mutations in *BRAF*, *RAS*, and *NF*. Mutations in these genes can facilitate escape from immune responses via several mechanisms: suppression of antigen expression, recruitment of regulatory T lymphocytes, and impaired dendritic cell maturation [27, 30]. In addition to immunoediting, *NRAS* mutations decrease Fas receptor expression and the susceptibility to Fas-mediated apoptosis in melanoma [31]. ALM lacks ultraviolet signatures, so it is plausible that ALM may possess a unique tumor microenvironment compared to other cutaneous melanoma subtypes. This could explain the differential effects of TILs on tumor progression among the subtypes, but further studies are necessary to validate this hypothesis.

This study has several limitations. First, it was performed in a single institute and the lymphocyte scoring method and analysis of the clinicopathological factors should be independently validated. However, we expect that the calculation of LS of intratumoral and peritumoral lymphocytes will be highly reproducible and that the data from different observers will agree closely. A second concern is that the mutation study was not fully performed in this study. We did not find a significant correlation between the presence of intra- or peritumoral lymphocytes and LS and genomic subtypes of cutaneous melanoma. However, BRAF mutations tended to be associated with the presence of peritumoral lymphocytes. According to previous studies [28, 32], cutaneous melanomas with mutations in *RAS* had less lymphocytic infiltration than those with the *BRAF* mutation. This intriguing issue may be resolved with additional studies on the effect of these mutations on intratumoral or peritumoral lymphocytes.

Finally, we conclude that the presence of peritumoral lymphocytes in cutaneous melanoma is a favorable prognostic factor that affects Breslow thickness and DFS. In patients with ALM, a high I-LS increases DFS more than just the presence of the lymphocytes. We suggest that the presence or absence of lymphocytes and the LS in both the intratumoral and peritumoral compartment should be included in the pathology report of cutaneous melanoma. Further studies should elucidate the correlation between intratumoral or peritumoral lymphocyte status and molecular subtypes of cutaneous melanoma.

## MATERIALS AND METHODS

### Study population

A total number of 732 consecutively diagnosed Korean patients who had undergone surgical treatment for melanoma from January 2006 to August 2015 in Severance Hospital, Seoul, Republic of Korea, were included. Patients who had a previous history of cutaneous melanoma, uveal melanoma, mucosal melanoma, melanoma *in situ*, tumors that were unsuitable for microscopic evaluation, or who had undergone neoadjuvant treatment were excluded. One-hundred and seventy-seven cases remained and were selected for the study.

Various clinical factors―age at time of surgery, sex, length of follow-up after surgery, recurrence, metastasis, and survival―were obtained from medical records. DFS time was estimated from the date of the surgical resection to the date of the first loco-regional or systemic metastasis, or death without any type of relapse. OS time was calculated from the date of the first surgical resection to the date of the last follow-up, or time of death regardless of cause. Whole-section slides of tissues stained with hematoxylin and eosin (H&E) were reviewed by two pathologists (C.K. Park and S.K. Kim). The pathological parameters including histological subtype, Clark level, Breslow thickness, ulceration, lymphovascular invasion, the presence or absence of sentinel lymph node metastasis, and the mutation analysis results performed at the time of diagnosis were obtained. Mitosis was counted in 10 high-power fields. This study was reviewed and approved by the Institutional Review Board at Severance Hospital (4-2015-0473).

### Evaluation and scoring of intratumoral and peritumoral lymphocytes

According to the previous studies [1, 19], intratumoral lymphocytes were defined as lymphocytes located in the stroma of the tumor mass or inside the tumor cell nests. Peritumoral lymphocytes were defined as lymphocytes surrounding the tumor mass. Applying this definition, lymphocytes were assessed as intratumoral and peritumoral and evaluated by two independent pathologists. The LS for each case was calculated as previously described [13], but, briefly, was defined as the sum of the lymphocyte distribution and density scores. Scores ranged from 0 to 6. The lymphocyte distribution score, which ranged from 0 to 3, was defined as follows: 0 = absence of lymphocytes within the tissue, 1 = presence of lymphocytes occupying < 25% of the tissue, 2 = presence of lymphocytes occupying 25 to 50% of the tissue, and 3 = presence of lymphocytes occupying > 50% of tissue. Lymphocyte density, which ranged from 0 to 3, was defined as follows: 0 = absent, 1 = mild, 2 = moderate, and 3 = severe. Based on this scoring method, the LS of the intratumoral compartment and the peritumoral compartment were calculated. We considered LS from 0 to 2 to be low and scores from 3 to 6 to be high.

### Mutation study

Genomic DNA was extracted from 10 μm sections cut from formalin-fixed paraffin-embedded (FFPE) tissue blocks using the Maxwell^®^ CSC DNA FFPE extraction kit (Promega, Wisconsin, USA) and the Maxwell^®^ CSC instrument. The genomic DNA was subjected to PCR amplification optimized for pyrosequencing analysis. The following program was used for PCR amplification: 95°C for 15 minutes; followed by 45 cycles of 94°C for 30 seconds, 59°C for 30 seconds, and 72°C for 30 seconds; and a final extension step of 72°C for 10 minutes. The sequences of the various PCR primers for *BRAF*, *KIT*, and *NRAS* genes are available on request.

After PCR analysis, single-stranded DNA suitable for pyrosequencing was prepared using the PyroMark Q24 MDx Vacuum Workstation (QIAGEN, Hilden, Germany). Pyrosequencing was performed in a PyroMark Q24 MDx Instrument according to the manufacturer's instructions. Sequencing data were analyzed using PyroMark Q24 MDx Software 2.0.

### Statistical analysis

Statistical calculations were performed with SPSS version 21.0 (SPSS Inc., Chicago, Illinois, USA). Chi-square tests were performed to evaluate the relationship between lymphocytes in the two compartments and clinicopathological parameters. DFS and OS were estimated with the Kaplan-Meier method with the log-rank test. Multivariate regression was analyzed using Cox proportional hazards model. Significance statements refer to *P* values of < 0.05 in two-tailed tests.

## SUPPLEMENTARY MATERIALS FIGURES AND TABLES


