# Supplementary material for: Clinicopathological significance of intratumoral and peritumoral lymphocytes and lymphocyte score based on the histologic subtypes of cutaneous melanoma
Source: Oncotarget. 2017 Jan 19;8(9):14759–69. doi: 10.18632/oncotarget.14736 (PMC5362441; doi:10.18632/oncotarget.14736)
Supplement: Supplementary file 1 [file oncotarget-08-14759-s001.pdf]

Clinicopathological significance of intratumoral and peritumoral lymphocytes and lymphocyte score based on the histologic subtypes of cutaneous melanoma

Supplementary Materials

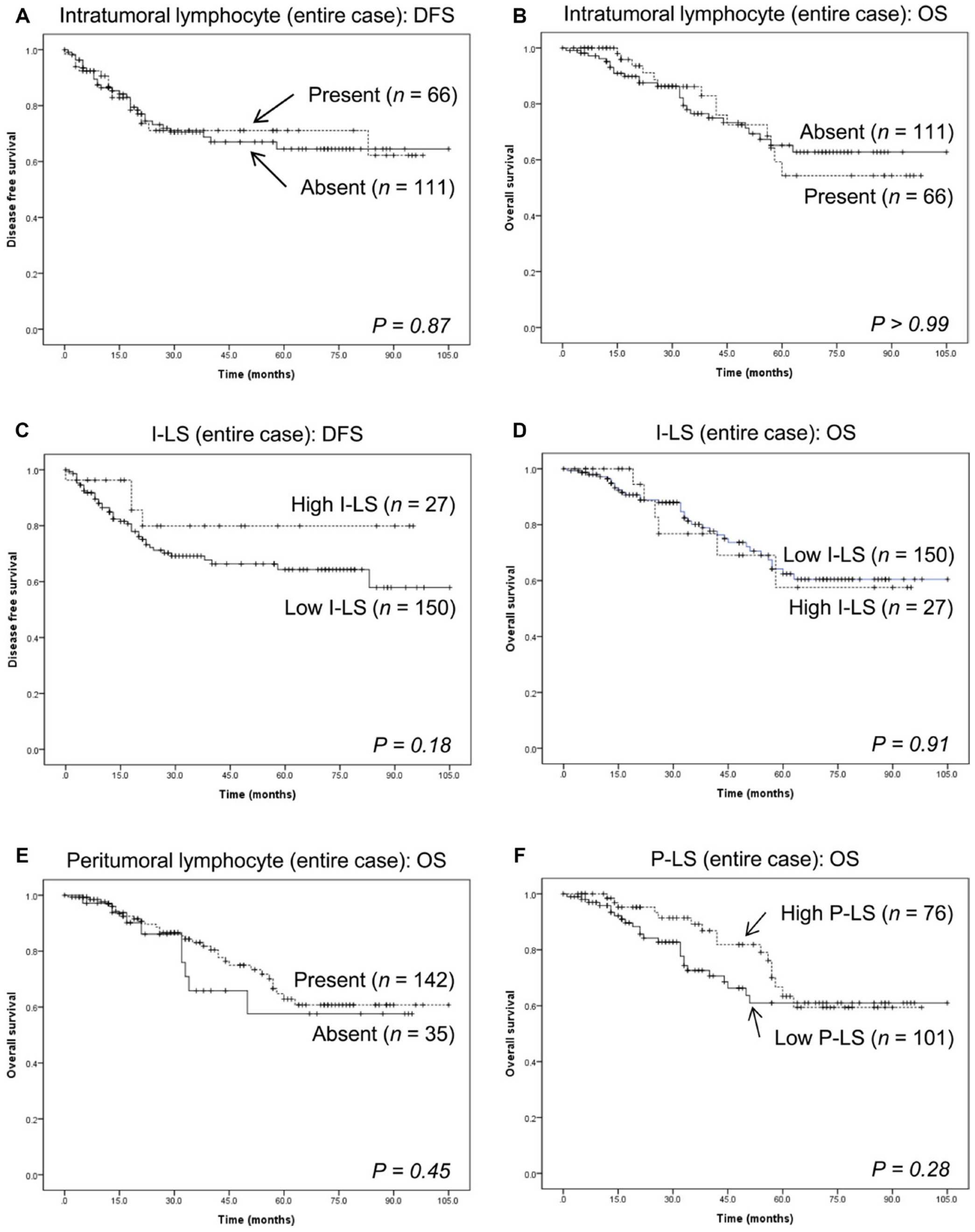

Supplementary Figure 1: Survival of 177 patients with cutaneous melanomas by Kaplan-Meier survival analysis. (**A**) and (**B**) DFS and OS compared to the presence or absence of intratumoral lymphocytes. (**C** and **D**) DFS and OS compared to the I-LS. (**E** and **F**) OS compared to the presence or absence of peritumoral lymphocytes and the P-LS.

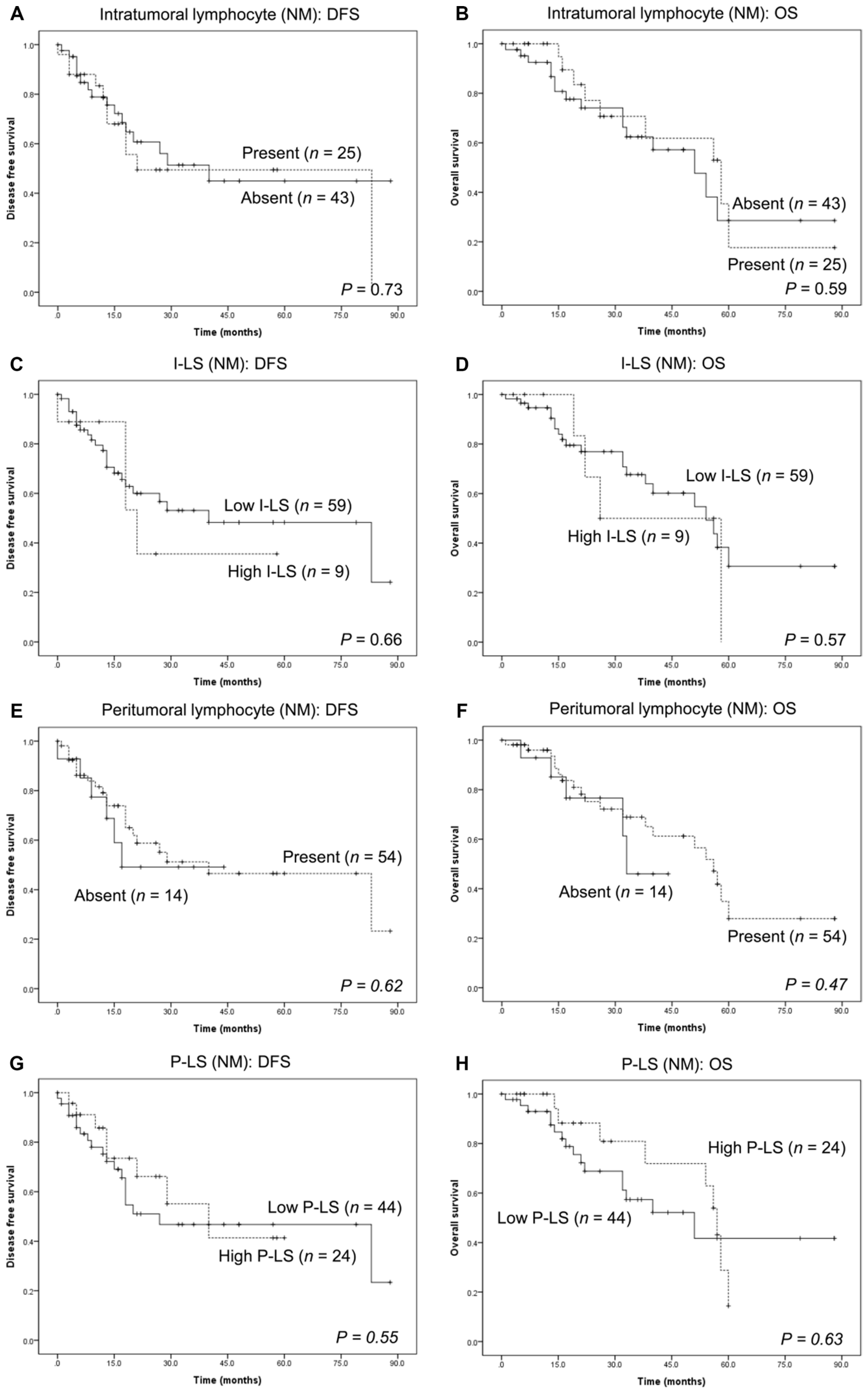

Supplementary Figure 2: Survival of 68 patients with nodular melanomas by Kaplan Meier analysis. DFS and OS compared to: (**A** and **B**) the presence or absence of intratumoral lymphocytes. (**C** and **D**) I-LS. (**E** and **F**) the presence or absence of peritumoral lymphocytes. (**G** and **H**) P-LS.

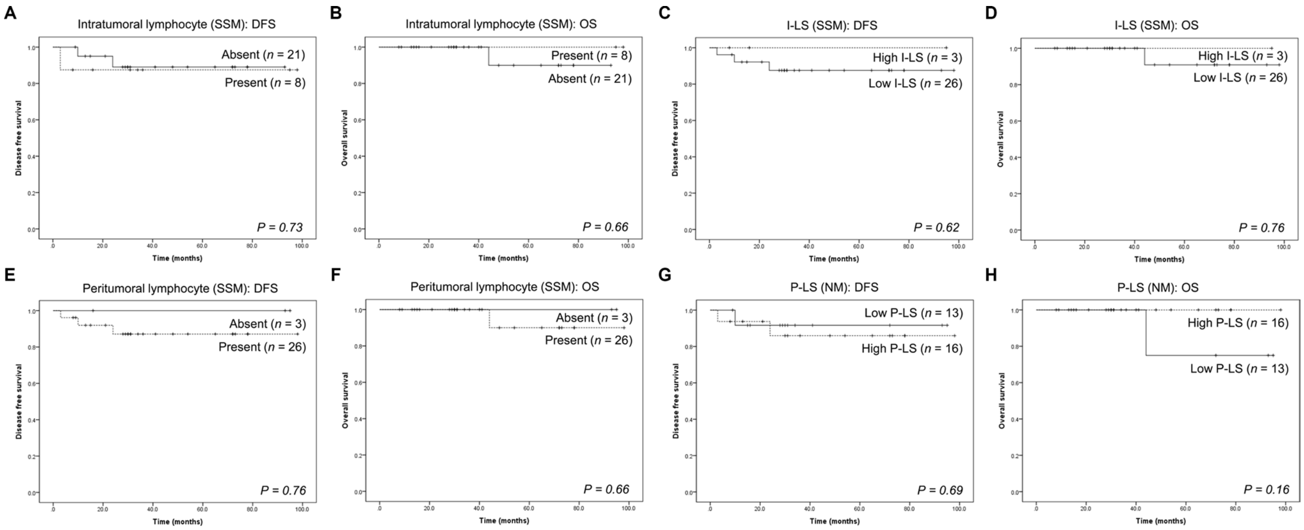

Supplementary Figure 3: Survival of 29 patients with superficial spreading melanomas by Kaplan Meier survival analysis. DFS and OS compared to: (**A** and **B**) the presence or absence of intratumoral lymphocytes. (**C** and **D**) I-LS. (**E** and **F**) the presence or absence of peritumoral lymphocytes. (**G** and **H**) P-LS.

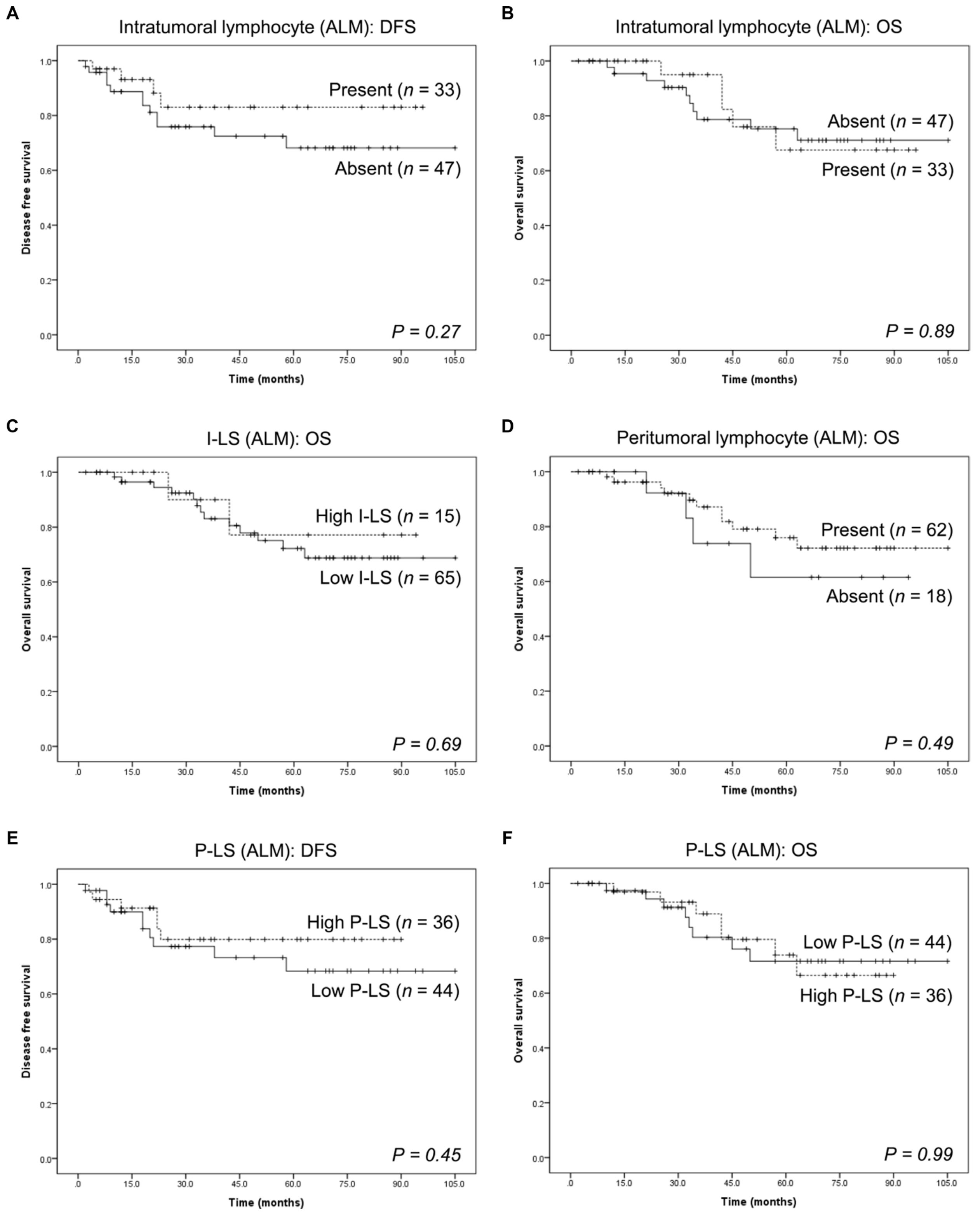

Supplementary Figure 4: Survival of 80 patients with acral lentiginous melanomas. (**A** and **B**) DFS and OS compared to the presence of intratumoral lymphocytes. (**C** and **D**) OS compared to I-LS and presence of peritumoral lymphocytes. (**E** and **F**) DFS and OS compared to P-LS.

Supplementary Table 1: Clinicopathological characteristics of 68 nodular melanomas according to intratumoral lymphocyte and lymphocyte score status

Category	Variables	Case No.	Intratumoral lymphocyte					Intratumoral lymphocyte score				
			Present (%)		Absent (%)		*P* value	High (%)		Low (%)		*P* value
			(*n* = 25)		(*n* = 43)			(*n* = 9)		(*n* = 59)		
Age	< 60 years	39	13	(52.0)	26	(60.5)	0.50	5	(55.6)	34	(57.6)	> 0.99
	≥ 60 years	29	12	(48.0)	17	(39.5)		4	(44.4)	25	(42.4)	
Gender	Male	34	14	(56.0)	20	(46.5)	0.45	6	(66.7)	28	(47.5)	0.48
	Female	34	11	(44.0)	23	(53.5)		3	(33.3)	31	(52.5)	
Clark level	II or III	4	3	(12.0)	1	(2.3)	0.14			4	(6.8)	> 0.99
	IV or V	64	22	(88.0)	42	(97.7)		9	(100	55	(93.2)	
Breslow thickness	≤ 1.00 mm	3	1	(4.0)	2	(4.7)	0.37	1	(11.1)	2	(3.4)	0.70
	1.01–2.00 mm	12	7	(28.0)	5	(11.6)		2	(22.2)	10	(16.9)	
	2.01–4.00 mm	15	4	(16.0)	11	(25.6)		2	(22.2)	13	(22.0)	
	> 4.00 mm	38	13	(52.0)	25	(58.1)		4	(44.4)	34	(57.6)	
Ulceration	Absent	39	15	(60.0)	24	(55.8)	0.74	8	(88.9)	31	(52.5)	0.07
	Present	29	10	(40.0)	19	(44.2)		1	(11.1)	28	(47.5)	
Mitosisa	≤ 5/10 HPFs	33	15	(60.0)	18	(41.9)	0.15	6	(66.7)	27	(45.8)	0.30
	> 5/10 HPFs	35	10	(40.0)	25	(58.1)		3	(33.3)	32	(54.2)	
LVI	Absent	57	19	(76.0)	38	(88.4)	0.31	7	(77.8)	50	(84.7)	0.63
	Present	11	6	(24.0)	5	(11.6)		2	(22.2)	9	(15.3)	
SLNMb	Absent	29	9	(42.9)	20	(54.1)	0.41	4	(50.0)	25	(50.0)	> 0.99
	Present	29	12	(57.1)	17	(45.9)		4	(50.0)	25	(50.0)	
Mutation statusc	*BRAF* mutant	10	5	(55.6)	5	(35.7)	0.42	3	(75.0)	7	(36.8)	0.28
	BRAF wild	13	4	(44.4)	9	(64.3)		1	(25.0)	12	(63.2)	
Recurrence/Metastasis	Absent	41	14	(56.0)	27	(62.8)	0.58	5	(55.6)	36	(61.0)	> 0.99
	Present	27	11	(44.0)	16	(37.2)		4	(44.4)	23	(39.0)	
Survival	Alive	43	16	(64.0)	27	(62.8)	0.92	5	(55.6)	38	(64.4)	0.72
	Expired	25	9	(36.0)	16	(37.2)		4	(44.4)	21	(35.6)	

HPF, high-power filed; LVI, lymphovascular invasion; SLNM, sentinel lymph node metastasis.
[a]Mean mitotic count of entire cases were 5.78/10HPFs.
[b]SLNM was Evaluated in 58 cases.
[c]Mutation study was performed in 23 cases. One NRAS mutant case belonged to *BRAF* wild group.

Supplementary Table 2: Clinicopathological characteristics of 68 nodular melanomas according to peritumoral lymphocyte and lymphocyte score status

Category	Variables	Case No.	Peritumoral lymphocyte				*P* value	Peritumoral lymphocyte score				*P* value
			Present (%) (*n* = 54)		Absent (%) (*n* = 14)			High (%) (*n* = 24)		Low (%) (*n* = 44)		
Age	< 60 years	39	31	(57.4)	8	(57.1)	0.99	14	(58.3)	25	(56.8)	0.90
	≥ 60 years	29	26	(42.6)	6	(42.9)		10	(41.7)	19	(43.2)	
Gender	Male	34	27	(50.0)	7	(50.0)	> 0.99	11	(45.8)	23	(52.3)	0.61
	Female	34	27	(50.0)	7	(50.0)		13	(54.2)	21	(47.7)	
Clark level	II or III	4	4	(7.4)			0.57	3	(12.5)	1	(2.3)	0.12
	IV or V	64	50	(92.6)	14	(100)		21	(87.5)	43	(97.7)	
Breslow thickness	≤ 1.00 mm	3	2	(3.7)	1	(7.1)	0.14	1	(4.2)	2	(4.5)	0.09
	1.01–2.00 mm	12	12	(22.2)				8	(33.3)	4	(9.1)	
	2.01–4.00 mm	15	13	(24.1)	2	(14.3)		5	(20.8)	10	(22.7)	
	> 4.00 mm	38	27	(50.0)	11	(78.6)		10	(41.7)	28	(63.6)	
Ulceration	Absent	39	31	(57.4)	8	(57.1)	0.99	17	(70.8)	22	(50.0)	0.10
	Present	29	23	(42.6)	6	(42.9)		7	(29.2)	22	(50.0)	
Mitosis[a]	≤ 5/10 HPFs	33	27	(50.0)	6	(42.9)	0.63	15	(62.5)	18	(40.9)	0.09
	> 5/10 HPFs	35	27	(50.0)	8	(57.1)		9	(37.5)	26	(59.1)	
LVI	Absent	57	47	(87.0)	10	(71.4)	0.22	20	(83.3)	37	(84.1)	> 0.99
	Present	11	7	(13.0)	4	(28.6)		4	(16.7)	7	(15.9)	
SLNM[b]	Absent	29	24	(52.2)	5	(41.7)	0.52	11	(61.1)	18	(45.0)	0.26
	Present	29	22	(47.8)	7	(58.3)		7	(38.9)	22	(55.0)	
Mutation status[c]	*BRAF* mutant	10	9	(50.0)	1	(20.0)	0.34	5	(50.0)	5	(38.5)	0.69
	BRAF wild	13	9	(50.0)	4	(80.0)		5	(50.0)	8	(61.5)	
Recurrence/Metastasis	Absent	41	33	(61.1)	8	(57.1)	0.79	16	(66.7)	25	(56.8)	0.43
	Present	27	21	(38.9)	6	(42.9)		8	(33.3)	19	(43.2)	
Survival	Alive	43	34	(63.0)	9	(64.3)	0.93	15	(62.5)	28	(63.6)	0.93
	Expired	25	20	(37.0)	5	(35.7)		9	(37.5)	16	(36.4)	

HPF, high-power filed; LVI, lymphovascular invasion; SLNM, sentinel lymph node metastasis.
[a]Mean mitotic count of entire cases were 5.78/10HPFs.
[b]SLNM was Evaluated in 58 cases.
[c]Mutation study was performed in 23 cases. One NRAS mutant case belonged to *BRAF* wild group.

Supplementary Table 3: Clinicopathological characteristics of 29 superficial spreading melanoma according to intratumoral lymphocyte and lymphocyte score status

Category	Variables	Case No.	Intratumoral lymphocyte				*P* value	Intratumoral lymphocyte score				*p*-value
			Present (%) (*n* = 8)		Absent (%) (*n* = 21)			High (%) (*n* = 3)		Low (%) (*n* = 26)		
Age	< 60 years	17	3	(37.5)	14	(66.7)	0.22			17	(65.4)	0.06
	≥ 60 years	12	5	(62.5)	7	(33.3)		3	(100)	9	(34.6)	
Gender	Male	11	4	(50.0)	7	(33.3)	0.43	1	(33.3)	9	(34.6)	0.54
	Female	18	4	(50.0)	14	(66.7)		2	(66.7)	17	(65.4)	
Clark level	II or III	16	3	(37.5)	13	(61.9)	0.41	1	(33.3)	15	(57.7)	0.57
	IV or V	13	5	(62.5)	8	(38.1)		2	(66.7)	11	(42.3)	
Breslow thickness	≤ 1.00 mm	20	6	(75.0)	14	(66.7)	0.79	2	(66.7)	18	(69.2)	0.92
	1.01–2.00 mm	8	2	(25.0)	6	(28.6)		1	(33.3)	7	(26.9)	
	2.01–4.00 mm	1			1	(4.8)				1	(3.8)	
Mitosis[a]	≤ 5/10 HPFs	26	7	(87.5)	19	(90.5)	> 0.99	3	(100)	23	(88.5)	> 0.99
	> 5/10 HPFs	3	1	(12.5)	2	(9.5)				3	(11.5)	
SLNM[b]	Absent	14	5	(100)	9	(90.0)	> 0.99	1	(100)	13	(92.9)	> 0.99
	Present	1			1	(10.0)				1	(7.1)	
Recurrence/Metastasis	Absent	26	7	(87.5)	19	(90.5)	> 0.99	3	(100)	23	(88.5)	> 0.99
	Present	3	1	(12.5)	2	(9.5)				3	(11.5)	
Survival	Alive	28	8	(100)	20	(95.2)	> 0.99	3	(100)	25	(96.2)	> 0.99
	Expired	1			1	(4.8)				1	(3.8)	

HPF, high-power filed; SLNM, sentinel lymph node metastasis.
[a]Mean mitotic count of entire cases were 5.78/10HPFs.
[b]SLNM was Evaluated in 15 cases.

Supplementary Table 4: Clinicopathological characteristics of superficial spreading melanoma according to peritumoral lymphocyte and lymphocyte score status

Category	Variables	Case No.	Peritumoral lymphocyte					Peritumoral lymphocyte score				
			Present (%) (*n* = 26)		Absent (%) (*n* = 3)		*P* value	High (%) (*n* = 16)		Low (%) (*n* = 13)		*P* value
Age	< 60 years	17	16	(61.5)	1	(33.3)	0.55	11	(68.8)	6	(46.2)	0.22
	≥ 60 years	12	10	(38.5)	2	(66.7)		5	(31.3)	7	(53.8)	
Gender	Male	11	10	(38.5)	1	(33.3)	> 0.99	8	(50.0)	3	(23.1)	0.25
	Female	18	16	(61.5)	2	(66.7)		8	(50.0)	10	(76.9)	
Clark level	II or III	16	15	(57.7)	1	(33.3)	0.57	10	(62.5)	6	(46.2)	0.38
	IV or V	13	11	(42.3)	2	(66.7)		6	(37.5)	7	(53.8)	
Breslow thickness	≤ 1.00 mm	20	18	(69.2)	2	(66.7)	0.92	11	(68.8)	9	(69.2)	0.64
	1.01–2.00 mm	8	7	(26.9)	1	(33.3)		4	(25.0)	4	(30.8)	
	2.01–4.00 mm	1	1	(3.8)				1	(6.3)			
Mitosis[a]	≤ 5/10 HPFs	26	23	(88.5)	3	(100)	> 0.99	16	(100)	10	(76.9)	0.08
	> 5/10 HPFs	3	3	(11.5)						3	(23.1)	
SLNM[b]	Absent	14	12	(92.3)	2	(100)	> 0.99	6	(85.7)	8	(100)	0.47
	Present	1	1	(7.7)				1	(14.3)			
Recurrence/Metastasis	Absent	26	23	(88.5)	3	(100)	> 0.99	14	(87.5)	12	(92.3)	> 0.99
	Present	3	3	(11.5)				2	(12.5)	1	(7.7)	
Survival	Alive	28	25	(96.2)	3	(100)	> 0.99	16	(100)	12	(92.3)	0.45
	Expired	1	1	(3.8)						1	(7.7)	

HPF, high-power filed; SLNM, sentinel lymph node metastasis.
[a]Mean mitotic count of entire cases were 5.78/10HPFs.
[b]SLNM was Evaluated in 15 cases.

Supplementary Table 5:Clinicopathological characteristics of 177 invasive cutaneous melanomas according to the combination of lymphocyte status of intratumoral and peritumoral compartment

Variables	Case No.	Absent in both compartment	Present in intratumoral comparment only	Present in peritumoral comparment only	Present in both compartment	*P*-value
		(*n* = 29, %)	(*n* = 6, %)	(*n* = 82, %)	(*n* = 60, %)	
Age						
< 60 years	95	17 (58.6)	1 (16.7)	50 (61.0)	27 (45.0)	0.07
≥ 60 years	82	12 (41.4)	5 (83.3)	32 (39.0)	33 (55.0)	
Gender						
Male	91	13 (44.8)	2 (33.3)	48 (58.5)	28 (46.7)	0.32
Female	86	16 (55.2)	4 (66.7)	34 (41.5)	32 (53.3)	
Histologic subtype						
ALM	80	15 (51.7)	3 (50.0)	32 (39.0)	30 (50.0)	0.08
NM	68	13 (44.8)	1 (16.7)	30 (36.6)	24 (40.0)	
SSM	29	1 (3.4)	2 (33.3)	20 (24.4)	6 (10.0)	
Clark level						
II or III	47	3 (10.3)	2 (33.3)	28 (34.1)	14 (23.3)	0.08
IV or V	130	26 (89.7)	4 (66.7)	54 (65.9)	46 (76.7)	
Breslow thickness						
≤ 1.00 mm	48	2 (6.9)	4 (66.7)	31 (37.8)	11 (18.3)	0.005
1.01–2.00 mm	47	6 (20.7)	1 (16.7)	19 (23.2)	21 (35.0)	
2.01–4.00 mm	33	7 (24.1)	1 (16.7)	15 (18.3)	10 (16.7)	
> 4.00 mm	49	14 (48.3)	0 (0.0)	17 (20.7)	18 (30.0)	
Ulceration						
Absent	126	18 (62.1)	5 (83.3)	67 (81.7)	36 (60.0)	0.02
Present	51	11 (37.9)	1 (16.7)	15 (18.3)	24 (40.0)	
Mitosis[a]						
≤ 5/10 HPFs	122	20 (69.0)	4 (66.7)	54 (65.9)	44 (73.3)	0.82
> 5/10 HPFs	55	9 (31.0)	2 (33.3)	28 (34.1)	16 (26.7)	
LVI						
Absent	162	24 (82.8)	5 (83.3)	79 (96.3)	54 (90.0)	0.11
Present	15	5 (17.2)	1 (16.7)	3 (3.7)	6 (10.0)	
SLNM[b]						
Absent	98	17 (70.8)	3 (75.0)	43 (75.4)	35 (67.3)	0.82
Present	39	7 (29.2)	1 (25.0)	14 (24.6)	17 (32.7)	
Mutation status[c]						
BRAF mutant	12	1 (9.1)	0 (0.0)	4 (30.8)	7 (38.9)	0.22
BRAF wild	30	10 (90.9)	0 (0.0)	9 (69.2)	11 (61.1)	
Recurrence /Metastasis						
Absent	131	17 (58.6)	5 (83.3)	64 (78.0)	45 (75.0)	0.21
Present	46	12 (41.4)	1 (16.7)	18 (22.0)	15 (25.0)	
Survival						
Alive	136	20 (69.0)	6 (100.0)	64 (78.0)	46 (76.7)	0.41
Expired	41	9 (31.0)	0 (0.0)	8 (22.0)	14 (23.3)	

ALM: acral lentiginous melanoma; NM: nodular melanoma; SSM: superficial spreading melanoma; HPF: high-power filed; LVI: lymphovascular invasion; SLNM: sentinel lymph node metastasis.
[a]Mean mitotic count of 177 cases were 5.78/10HPFs.
[b]SLNM was Evaluated in 137 cases.
[c]Mutation study was performed in 42 cases. Two NRAS mutant cases belonged to *BRAF* wild group.

Supplementary Table 6: Clinicopathological characteristics of 177 invasive cutaneous melanomas according to the combination of lymphocyte score status of intratumoral and peritumoral compartment

Variables	Case No.	Low LS in both compartment (*n* = 92, %)	High I-LS only (*n* = 9, %)	High P-LS only (*n* = 58, %)	High LS in both compartment (*n* = 18, %)	*P*-value
Age						
< 60 years	95	52 (56.5)	3 (33.3)	33 (56.9)	7 (38.9)	0.31
≥ 60 years	82	40 (43.5)	6 (66.7)	25 (43.1)	11 (61.1)	
Gender						
Male	91	54 (58.7)	2 (22.2)	28 (48.3)	7 (38.9)	0.32
Female	86	38 (41.3)	7 (77.8)	30 (51.7)	11 (61.1)	
Histologic subtype						
ALM	80	41 (44.6)	3 (33.3)	24 (41.4)	12 (66.7)	0.13
NM	68	40 (43.5)	4 (44.4)	19 (32.8)	5 (27.8)	
SSM	29	11 (12.0)	2 (22.2)	15 (25.9)	1 (5.6)	
Clark level						
II or III	47	15 (10.3)	2 (22.2)	23 (39.7)	7 (38.9)	0.009
IV or V	130	77 (89.7)	7 (77.8)	35 (60.3)	11 (61.1)	
Breslow thickness						
≤ 1.00 mm	48	16 (17.4)	4 (44.4)	23 (39.7)	5 (27.8)	0.003
1.01–2.00 mm	47	20 (21.7)	4 (44.4)	19 (32.8)	4 (22.2)	
2.01–4.00 mm	33	20 (21.7)	0 (0.0)	7 (12.1)	6 (33.3)	
> 4.00 mm	49	36 (39.1)	1 (11.1)	9 (15.5)	3 (16.7)	
Ulceration						
Absent	126	60 (65.2)	8 (88.9)	46 (79.3)	12 (66.7)	0.17
Present	51	32 (34.8)	1 (11.1)	11 (20.7)	6 (33.3)	
Mitosis[a]						
≤ 5/10 HPFs	122	56 (60.9)	6 (66.7)	44 (75.9)	16 (88.9)	0.06
> 5/10 HPFs	55	36 (39.1)	3 (33.3)	14 (24.1)	2 (11.1)	
LVI						
Absent	162	84 (91.3)	8 (88.9)	53 (91.4)	17 (94.4)	0.96
Present	15	8 (8.7)	1 (11.1)	5 (8.6)	1 (5.6)	
SLNMb						
Absent	98	52 (68.4)	5 (71.4)	31 (81.6)	10 (62.5)	0.41
Present	39	24 (31.6)	2 (28.6)	7 (18.4)	6 (37.5)	
Mutation status[c]						
BRAF mutant	12	5 (21.7)	1 (100.0)	4 (28.6)	2 (50.0)	0.27
BRAF wild	30	18 (78.3)	0 (0.0)	10 (71.4)	2 (50.0)	
Recurrence / Metastasis						
Absent	131	65 (70.7)	6 (66.7)	43 (74.1)	17 (94.4)	0.20
Present	46	27 (29.3)	3 (33.3)	15 (25.9)	1 (5.6)	
Survival						
Alive	136	69 (75.0)	7 (77.8)	46 (79.3)	14 (77.8)	0.94
Expired	41	23 (25.0)	2 (22.2)	12 (20.7)	4 (22.2)	

ALM: acral lentiginous melanoma; NM: nodular melanoma; SSM: superficial spreading melanoma; HPF: high-power filed; LVI: lymphovascular invasion; SLNM: sentinel lymph node metastasis.

[a]Mean mitotic count of 177 cases were 5.78/10HPFs.

[b]SLNM was Evaluated in 137 cases.

[c]Mutation study was performed in 42 cases. Two NRAS mutant cases belonged to *BRAF* wild group.

Supplementary Table 7: Clinicopathological characteristics of 80 acral lentiginous melanomas according to the combination of lymphocyte status of intratumoral and peritumoral compartment

Variables	Case No.	Absent in both compartment	Present in intratumoral comparment only	Present in peritumoral comparment only	Present in both compartment	*P*-value
		(*n* = 15, %)	(*n* = 3, %)	(*n* = 32, %)	(*n* = 30, %)	
Age						
< 60 years	39	9 (60.0)	0 (0.0)	18 (56.3)	12 (40.0)	0.15
≥ 60 years	41	6 (40.0)	3 (100.0)	14 (43.8)	18 (60.0)	
Gender						
Male	39	5 (33.3)	1 (33.3)	19 (59.4)	14 (46.7)	0.36
Female	41	10 (66.7)	2 (66.7)	13 (40.6)	16 (53.3)	
Clark level						
II or III	27	3 (20.0)	1 (33.3)	14 (43.8)	9 (30.0)	0.41
IV or V	53	12 (80.0)	2 (66.7)	18 (56.3)	21 (70.0)	
Breslow thickness						
≤ 1.00 mm	25	2 (13.3)	1 (33.3)	15 (46.9)	7 (23.3)	0.49
1.01–2.00 mm	27	5 (33.3)	1 (33.3)	9 (28.1)	12 (40.0)	
2.01–4.00 mm	17	5 (33.3)	1 (33.3)	5 (15.6)	6 (20.0)	
> 4.00 mm	11	3 (20.0)	0 (0.0)	3 (9.4)	5 (16.7)	
Ulceration						
Absent	58	10 (66.7)	2 (66.7)	30 (93.8)	16 (53.3)	0.004
Present	22	5 (33.3)	1 (33.3)	2 (6.3)	14 (46.7)	
Mitosis[a]						
≤ 5/10 HPFs	63	13 (86.7)	2 (66.7)	24 (75.0)	24 (80.0)	0.77
> 5/10 HPFs	17	2 (13.3)	1 (33.3)	8 (25.0)	6 (20.0)	
LVI						
Absent	76	13 (86.7)	3 (100.0)	31 (96.9)	29 (96.7)	0.43
Present	4	2 (13.3)	0 (0.0)	1 (3.1)	1 (3.3)	
SLNM[b]						
Absent	55	11 (91.7)	2 (100.0)	20 (90.9)	22 (78.6)	0.50
Present	9	1 (8.3)	0 (0.0)	2 (9.1)	6 (21.4)	
Mutation status[c]						
BRAF mutant	2	0 (0.0)	0 (0.0)	0 (0.0)	2 (28.6)	0.20
BRAF wild	15	6 (100.0)	0 (0.0)	4 (100.0)	5 (71.4)	
Recurrence/ Metastasis						
Absent	64	8 (53.3)	3 (100.0)	27 (84.4)	26 (86.7)	0.04
Present	16	7 (46.7)	0 (0.0)	5 (15.6)	4 (13.3)	
Survival						
Alive	65	11 (73.3)	3 (100.0)	26 (81.3)	25 (83.3)	0.71
Expired	15	4 (26.7)	0 (0.0)	6 (18.8)	5 (16.7)	

HPF: high-power field; LVI: lymphovascular invasion; SLNM: sentinel lymph node metastasis.
[a]Mean mitotic count of 177 cases were 5.78/10HPFs.
[b]SLNM was Evaluated in 62 cases.
[c]Mutation study was performed in 17 cases. One *NRAS* mutant case belonged to *BRAF* wild group.

Supplementary Table 8: Clinicopathological characteristics of 80 acral lentiginous melanomas according to the combination of lymphocyte score status of intratumoral and peritumoral compartment

Variables	Case No.	Low LS in both compartment (*n* = 41, %)	High I-LS only (*n* = 3, %)	High P-LS only (*n* = 24, %)	High LS in both compartment (*n* = 12, %)	*P*-value
Age						
< 60 years	39	24 (58.5)	0 (0.0)	10 (41.7)	5 (41.7)	0.16
≥ 60 years	41	17 (41.5)	3 (100.0)	14 (58.3)	7 (58.3)	
Gender						
Male	39	25 (61.0)	0 (0.0)	9 (37.5)	5 (41.7)	0.08
Female	41	16 (39.0)	3 (100.0)	15 (62.5)	7 (58.3)	
Clark level						
II or III	27	9 (22.0)	1 (33.3)	10 (41.7)	7 (58.3)	0.09
IV or V	53	32 (78.0)	2 (66.7)	14 (58.3)	5 (41.7)	
Breslow thickness						
≤ 1.00 mm	25	8 (19.5)	1 (33.3)	11 (45.8)	5 (41.7)	0.20
1.01–2.00 mm	27	14 (34.1)	2 (66.7)	8 (33.3)	3 (25.0)	
2.01–4.00 mm	17	10 (24.4)	0 (0.0)	3 (12.5)	4 (33.3)	
> 4.00 mm	11	9 (22.0)	0 (0.0)	2 (8.3)	0 (0.0)	
Ulceration						
Absent	58	30 (73.2)	3 (100.0)	19 (79.2)	6 (50.0)	0.19
Present	22	11 (26.8)	0 (0.0)	5 (20.8)	6 (50.0)	
Mitosis[a]						
≤ 5/10 HPFs	63	32 (78.0)	2 (66.7)	18 (75.0)	11 (91.7)	0.64
> 5/10 HPFs	17	9 (22.0)	1 (33.3)	6 (25.0)	1 (8.3)	
LVI						
Absent	76	39 (95.1)	3 (100.0)	22 (91.7)	12 (100.0)	0.72
Present	4	2 (4.9)	0 (0.0)	2 (8.3)	0 (0.0)	
SLNM[b]						
Absent	55	29 (87.9)	2 (100.0)	16 (94.1)	8 (66.7)	0.17
Present	9	4 (12.1)	0 (0.0)	1 (5.9)	4 (33.3)	
Mutation status[c]						
BRAF mutant	2	1 (10.0)	0 (0.0)	1 (16.7)	0 (0.0)	0.86
BRAF wild	15	9 (90.0)	0 (0.0)	5 (83.3)	1 (100.0)	
Recurrence /Metastasis						
Absent	64	31 (75.6)	3 (100.0)	18 (75.0)	12 (100.0)	0.20
Present	16	10 (24.4)	0 (0.0)	6 (25.0)	0 (0.0)	
Survival						
Alive	65	33 (80.5)	3 (100.0)	19 (79.2)	10 (83.3)	0.85
Expired	15	8 (19.5)	0 (0.0)	5 (20.8)	2 (16.7)	

HPF: high-power field; LVI: lymphovascular invasion; SLNM: sentinel lymph node metastasis.
[a]Mean mitotic count of 177 cases were 5.78/10HPFs.
[b]SLNM was Evaluated in 62 cases.
[c]Mutation study was performed in 17 cases. One NRAS mutant case belonged to *BRAF* wild group.

SupplementaryTable 9: Multivariate analysis of 80 acral lentiginous melanomas of the impact of variable clinicopathologic factors on recurrence/metastasis and survival

Category	Variables	Recurrence/Metastasis		Survival	
		HR (95% CI)	*P* value	HR (95% CI)	*P* value
Age	< 60 years	1		1	
	≥ 60 years	0.868 (0.232–3.242)	0.83	5.404 (0.293–99.564)	0.26
Gender	Female	1		1	
	Male	1.902 (0.376–9.626)	0.44	4.265 (0.412–44.149)	0.22
Clark level	II or III	1		1	
	IV or V	1.303 (0.161–10.523)	0.80	2.702 (0.092–79.671)	0.57
Breslow thickness	≤ 1.00 mm	1		1	
	1.01–2.00 mm	1.839 (0.163–20.768)	0.62	0.433 (0.003–57.629)	0.74
	2.01–4.00 mm	2.019 (0.115–35.395)	0.63	21.829 (0.129–3,681.181)	0.24
	> 4.00 mm	3.795 (0.109–131.56)	0.46	353.96 (0.206–609,393.83)	0.12
Ulceration	Absent	1		1	
	Present	0.566 (0.071–4.546)	0.59	1.669 (0.154–18.131)	0.67
Mitosis	≤ 5/10 HPFs	1		1	
	> 5/10 HPFs	1.137 (0.187–6.907)	0.89	0.473 (0.050–4.497)	0.51
LVI	Absent	1		1	
	Present	1.161 (0.056–24.256)	0.92	0.944 (0.005–175.826)	0.98
SLNM	Absent	1		1	
	Present	1.828 (0.160–20.921)	0.63	1.292 (0.102–16.359)	0.84
Intratumoral lymphocyte	Absent	1		1	
	Present	1.330 (0.213–8.293)	0.76	0.197 (0.007–5.609)	0.34
Peritumoral lymphocyte	Absent	1		1	
	Present	0.416 (0.062–2.791)	0.37	0.118 (0.002–6.899)	0.30
I–LS	Low	1		1	
	High	0.000 (0.000–1.63E+240)	0.97	0.699 (0.028–17.436)	0.83
P–LS	Low	1		1	
	High	1.829 (0.196–17.072)	0.60	21.908 (0.222–2,161.884)	0.19

HR, hazard ratio; CI, confidence interval; HPF, high-power filed; LVI: lymphovascular invasion; SLNM, sentinel lymph node metastasis; I-LS, intratumoral lymphocyte score; P-LS, peritumoral lymphocyte score.
